# Exenatide induces autophagy and prevents the cell regrowth in HepG2 cells

**DOI:** 10.17179/excli2019-1415

**Published:** 2019-07-22

**Authors:** Gabriele Catyana Krause, Kelly Goulart Lima, Vitor Levorse, Gabriela Viegas Haute, Rodrigo Benedetti Gassen, Maria Cláudia Garcia, Leonardo Pedrazza, Márcio Vinícius Fagundes Donadio, Carolina Luft, Jarbas Rodrigues de Oliveira

**Affiliations:** 1 Laboratório de Pesquisa em Biofísica Celular e Inflamação, Pontifícia Universidade Católica do Rio Grande do Sul (PUCRS), Porto Alegre, Rio Grande do Sul, Brazil; 2 Laboratório de Imunologia Celular e Molecular, Hospital São Lucas, Pontifícia Universidade Católica do Rio Grande do Sul (PUCRS), Porto Alegre, Rio Grande do Sul, Brazil; 3 Ubiquitylation and Cell Signaling Lab. IDIBELL, Department de Ciències Fisiològiques, Universitat de Barcelona, L'Hospitalet de Llobregat - Barcelona, Spain; 4 Laboratório de Atividade Física em Pediatria, Centro Infant, Pontifícia Universidade Católica do Rio Grande do Sul (PUCRS), Porto Alegre, Rio Grande do Sul, Brazil

**Keywords:** exenatide, HepG2, hepatocellular carcinoma, autophagy, regrowth, mTOR

## Abstract

The incidence of hepatocellular carcinoma (HCC) keeps rising year by year, and became the second leading cause of cancer-related death. Some studies have found that liraglutide, a GLP-1 analog, may decrease the tumor cells proliferation. Due to this, the aim of this work is to investigate the antiproliferative potential of exenatide, another GLP-1 analog. Cell proliferation was assessed by direct count with Trypan blue dye exclusion. Flow cytometry was used to determinate autophagy and nuclear staining. Morphometric analysis was used to verify senescence and apoptosis. The mechanism that induced cell growth inhibition was analyzed by Western Blot. Treatment with exenatide significantly decreases cell proliferation and increases autophagy, both in relation to control and liraglutide. In addition, mTOR inhibition was greater in cells treated with exenatide. In relation to chronic treatment, exenatide does not allow cellular regrowth by preventing some resistance mechanism that the cells can acquire. These results suggest that exenatide has a potent anti-proliferative activity via mTOR modulation and, among the GLP-1 analogs tested, could be in the future an alternative for HCC treatment.

## Introduction

Hepatocellular cancer is one of the most incidental and lethal types of solid tumor in the world and the third leading cause of cancer-related death (Waller et al., 2015[27]; Ge and Huang, 2015[9]). Several factors may contribute to the development of HCC, although hepatitis B and C are responsible for about 80 % of cases (Davis et al., 2008[6]).

Without presenting symptomatology in the initial phase, most HCCs are already at an advanced stage with no opportunity of radical operations upon diagnosis and, with cancer progression and aggravation of liver dysfunctions, systemic drug treatment becomes inefficient resulting in a worse prognosis for HCC (Ge and Huang, 2015[9]). In fact, standard systemic chemotherapy is typically not effective for HCC treatment, and until now sorafenib seems to be the only available treatment for advanced stages (Le Grazie et al., 2017[15]), which makes the search for new chemotherapeutics increasingly important and necessary.

Glucagon-like peptide-1 (GLP-1), an intestinal incretin produced in L-cells and release in response to meal intake, works by promoting the secretion of insulin and decreasing plasma glucose. GLP-1 analogs act as anti-glycemic hormone, inducing the insulin release from pancreatic β cells. Studies have shown its beneficial effects in some diseases, including decreased appetite and gastric emptying, immune regulation, and neuronal protection (Abu-Hamdah et al., 2009[1]; Chai et al., 2014[3]; Seufert and Gallwitz, 2014[23]; Ussher and Drucker, 2014[26]). 

Exendin-4 is a peptide with 39 amino acids found in the saliva of the lizard Gila monster (Eng et al., 1992[8]). It has about 50 % homology to human GLP-1 and a half-life greater than observed for GLP-1 (>10 min) (Nielsen et al., 2004[18]). The main difference is the presence of glycine in the second amino acid of exanatide-4, which is resistant to dipeptidyl peptidase-4 (DPP-4) cleavage, resulting in a half-life of 30 minutes in humans. Exenatide is a synthetic form of exendin-4 and was the first FDA-approved GLP-1 drug in 2005. Evidence has shown the potential benefits of exendin-4 in modulating lipid metabolism in a murine model of hepatic steatosis and in human hepatocyte (Yoo et al., 2018[28]; Gupta et al., 2010[10]). Moreover, one study demonstrated that GLP-1 based agents, liraglutide and exendin-4, could reduce steatosis by enhancing autophagy (Sharma et al., 2011[24]).

Thus, considering that we have previously shown that liraglutide, a GLP-1 analog, presents chemotherapeutic effects for cancer by inducing autophagy and senescence in hepatocarcinoma cells (Krause et al., 2017[14]), the aim of this study was to compare the antiproliferative effects of exenatide and liraglutide.

## Materials and Methods

### Cells and treatment

Human hepatocellular carcinoma HepG2 cell line was acquired from the cell bank of Federal University of Rio de Janeiro (Rio de Janeiro, Brazil). Cells were grown in Dulbecco's Modified Eagle medium (DMEM; Gibco, USA) supplemented with 10 % fetal bovine serum (FBS; Gibco, USA) FBS, 1 % penicillin (100 units/mL) and streptomycin (100 µg/mL) (Invitrogen, USA), 2 g/L HEPES buffer and 3,7 g/L NaHCO_3_ in humidified atmosphere of 5 % CO_2_ at 37 °C.

Exenatide (Byetta, Baxter Pharmaceutical Solutions, USA) and liraglutide (Victoza, Novo Nordisk A/S, Denmark) were used at a final concentration of 15 µM (Krause et al., 2017[14]). HepG2 cells were incubated and the analyses were performed 48 h after treatment. All experiments were performed five times.

### Cell proliferation

HepG2 cells were seeded at a density of 2.5×10^4^ cells/well into 96-well plates and treated with exenatide or liraglutide (15 µM) for 48 h. The cell number was counted in a Neubauer chamber by Trypan blue dye exclusion. The cellular viability was evaluated by mixing 25 μL of cell suspension and 25 μL of 0.4 % Trypan blue stain solution (Sigma-Aldrich, USA). Blue cells were counted as dead cells. Three replicate wells were used at each concentration tested, including control.

### Cytotoxicity

The analysis of the enzyme lactate dehydrogenase (LDH) was used to assess treatment cytotoxicity in HepG2 cells, since the LDH released from the cytoplasm into the cell culture medium is an evidence of cellular damage (membrane disruption). Lactate dehydrogenase assay (Labtest Diagnóstica, Brazil) was performed in both supernatants and cell lysate following manufacturer's instructions. The LDH concentration was determinated spectrophotometrically at 492 nm. For the control of cell lysis, DMEM with 5 % Tween solution was used. For the evaluation of the enzymatic content, the sum of the LDH amount in the supernatant and the cell lysate was considered 100 %.

### Nuclear morphometric analysis (NMA)

To evaluate apoptosis and senescence, the size and irregularity of the cellular nuclei were analyzed. Cells were seeded in 24-well plates (10×10^3^ cells/well), treated with 15 µM of exenatide and 20 µM of cisplatin. After 48 h of incubation, the cells were fixed with 4 % paraformaldehyde for 2 h. Next, fixed cells were permeabilized with 0.3 % Triton X-100 in phosphate buffered saline (PBS) for 30 min and stained with 300 nM of 4´,6-diamidino-2-phenylindole (DAPI) for 2 min in a dark condition at room temperature. Cells were photographed with a fluorescence microscope (IX71, Olympus) and the nuclear morphometry was evaluated using the Image Pro Plus 6.0 software (IPP 6 - Media Cybernetics, USA). The nuclear morphological changes were classified as normal, small and regular, large and regular, or irregular.

### Autophagy analysis

The formation of acidic vesicular organelles (AVO's) is a morphological characteristic of autophagy. We quantified AVO's by Acridine Orange staining, in which the acid compartments display orange fluorescence, an autophagic marker. Briefly, the cells were seeded into 96-well plates at a density of 2.5×10^4^ cells per well and treated with 15 µM of exenatide and 200 nM of rapamycin for 48 h. The Acridine Orange marked cells were evaluated with FACSCanto II flow cytometer (BD Bioscience, USA) and analyzed using FlowJo 10.0.8 software (Tree Star Inc., USA).

### mTOR activation

In order to evaluate the exenatide activity on mammalian target of rapamycin (mTOR), cells were treated with insulin 200 nM (positive control for mTOR activation), rapamycin 200 nM (positive control for mTOR inhibition) and GLP-1 analogs. In order to determine the cell number, cells were counted with a Neubauer chamber. The cellular viability was evaluated by mixing 25 μL of cell suspension and 25 μL of 0.4 % Trypan blue stain solution (Sigma-Aldrich, USA). Blue cells were counted as dead cells.

### mTOR expression

For the Western Blot analysis, cells were rinsed twice with PBS and lysed with Triton lysis buffer with protease inhibitor for protein extraction. Aliquots from each sample containing equal amounts of protein were run on a SDS page gel, and transferred to a nitrocellulose membrane. Next, membranes were blocked with Tris buffered saline containing 0.05 % Tween-20 and 5 % non-fat dry milk and incubated overnight at 4 ºC with primary antibodies against mTOR (1:500 dilution; Cell Signaling Technology, USA) and GAPDH (1:1000 dilution; Thermo Fisher Scientific, USA). Then, the membranes were incubated for 1 h with the appropriate horseradish peroxidase-conjugated secondary antibody (Goat anti-Rabbit, dilution 1:2000, Cell Signaling Technology, USA; and Rabbit anti-Mouse, dilution 1:2000, Thermo Fisher Scientific, USA) at room temperature. Values of proteins were normalized to GAPDH.

### Treatment resistance analysis

Cumulative population doubling (CPD) assay was used to evaluate the proliferation rate and the regrowth of HepG2 cells after exenatide (15 µM), liraglutide (15 µM) or cisplatin (20 µM) treatment. The cells were seeded (5 x 10^5^) in a 6-well plate and harvested for 48 h. After, cells were seeded with a cell density of 12 x 10^4^, 9 x 10^4^, 6 x 10^4^ and 3 x 10^4^ cells per well in 24-well plates and incubated for 24 h. The cells were retreated as previously described, followed by cell counting. The number of viable cells was determined by Trypan blue stain solution (Sigma-Aldrich, USA), using a hemocytometer under a light microscope (Nikon Optiphot, Japan). The cells were counted in the second, third, fourth and fifth day after seeding. Population doubling (PD) of each interval was determined according to the formula PD = [log N(t) - log N(to)]/log 2, where N(t) is the number of cells at the time count, and N(to) is the number of cells seeded. The sum of PDs was then plotted against time of culture.

### Statistical analysis

The normality of the data was tested using the Shapiro-Wilk test. Data were analyzed using a one way ANOVA followed by Tukey multiple comparison test using Statistical Package for the Social Sciences Version 13.0 (SPSS, Inc., USA). A value of *p*<0.05 was considered as statistically significant.

## Results

**Figure 1:**
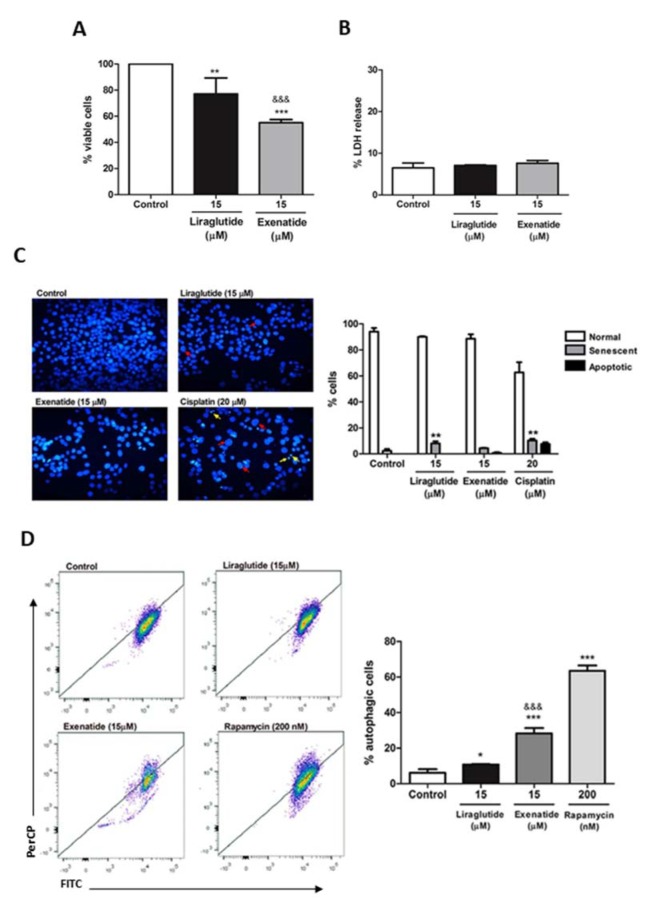
Effect of exenatide and liraglutide on cell proliferation, cytotoxicity, nuclear morphometry and autophagy of HepG2 cells. (A) HepG2 cells were treated with liraglutide (15 µM) or exenatide (15 µM) for 48 h. Cell viability was assessed by direct cell counting. Results are expressed as percentage of cells in relation to control. (B) Percentage of LDH release by HepG2 cells after treatment with liraglutide (15 μM) or exenatide (15 μM). Results are expressed as percentage of LDH release into the supernatant. (C) Representative images of nuclei from cell control and cells exposed to liraglutide (15 μM), exenatide (15 μM), and cisplatin (20 μM) for positive control. Red arrows indicate senescent nuclei and yellow arrows apoptotic nuclei. (D) DAPI-stained nuclei were analyzed for size and irregularity, and the percentage of senescent cells is show. (E) Representative flow cytometry plots in autophagy FITC (x axis) / PerCP Cy5 (y axis). (F) Cells were exposed to liraglutide (15 μM), exenatide (15 μM) and, rapamycin (200 nM). Results are expressed as percentage of autophagic cells. Data represent the mean ± SD (*n* = 3-5) (*p < 0.05, **p < 0.01, ***p < 0.001 vs control) (^&&& ^*p* < 0.001 vs liraglutide).

First, we investigated which of the treatments showed better antiproliferative effect. We observed that exenatide treatment was able to decrease viable cells by around 45 %, whereas liraglutide demonstrated a 23 % of proliferation decrease (Figure 1A(Fig. 1)). In order to assess whether this growth decrease was caused by cytotoxicity, the LDH release was evaluated. There was no significant difference between treated and control groups, demonstrating that viability decrease was not due to necrosis, suggesting that another mechanism may be involved in this response (Figure 1B(Fig. 1)). 

Senescence and apoptosis are two know mechanisms of anticancer drugs. For this reason, they were evaluated as a possible cause of the decreased cell proliferation observed. In the NMA test, it is possible to evaluate nuclear morphometric parameters that enable the identification of these cellular processes. In Figure 1C(Fig. 1), red arrows indicate senescent nuclei and yellow arrows apoptotic nuclei. Exenatide treatment did not demonstrate an increase of senescent or apoptotic cells, unlike liraglutide treatment, which demonstrated senescence induction, indicating that the drugs may act on different routes. Cisplatin was used as a positive control (Figure 1C and 1D(Fig. 1)). 

Autophagy is another well-known mechanism of cell proliferation decreased in cancer, and, for this reason, we investigated if exenatide and liraglutide were able to induce autophagy in HCC cells. Our results demonstrate that exenatide treatment significantly increases autophagy, both in comparison to the control and in comparison to liraglutide treatment. Rapamycin was used as a positive control (Figure 1E and 1F(Fig. 1)). 

**Figure 2:**
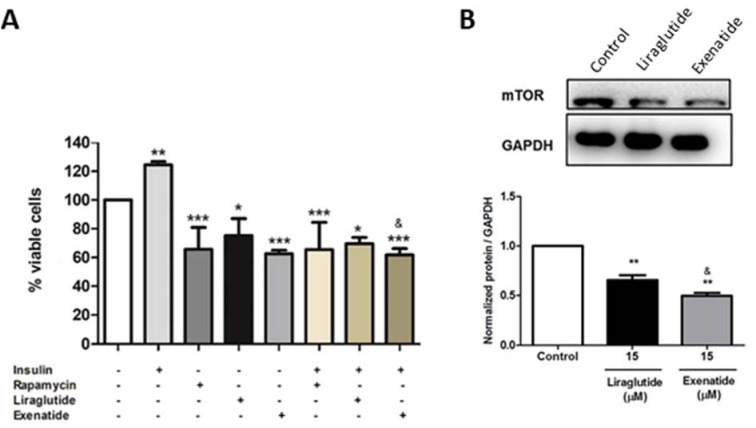
Effect of GLP-1 analogs on mTOR activation and protein expression. (A) HepG2 cells were treated with insulin (200 nM), rapamycin (200 nM), liraglutide (15 μM) or exenatide (15 μM) for 48 h. Cell viability was assessed by direct cell counting. Results are expressed as percentage of cells in relation to control. Data represent the mean ± SD (*n* =5) (*p < 0.05 vs control, **p < 0.01 vs control, ***p < 0.001 vs control) (^&^ *p* < 0.05 vs liraglutide). (B) mTOR expression on HepG2 cells after treatment for 48h with liraglutide (15 μM) or exenatide (15 μM). Results are expressed as normalized protein/GAPDH. Data represent the mean ± SD (**p < 0.01 vs control) (^&^ *p* < 0.05 vs liraglutide).

Next, we tried to verify if the decrease in cell proliferation by exenatide was related to the modulation of mTOR signaling. Thus, the cells were pre-treated or not with insulin, rapamycin, liraglutide, and exenatide. Our results showed that exenatide is able to inhibit insulin stimulation, as well as rapamycin and liraglutide, in a more pronounced way than liraglutide, suggesting that one possible mechanism of action is through the mTOR pathway (Figure 2A(Fig. 2)). To confirm these findings, we have also evaluated the mTOR protein expression and results have shown a decrease in the treated groups, with the exenatide effect more potent than liraglutide (Figure 2B(Fig. 2)).

**Figure 3:**
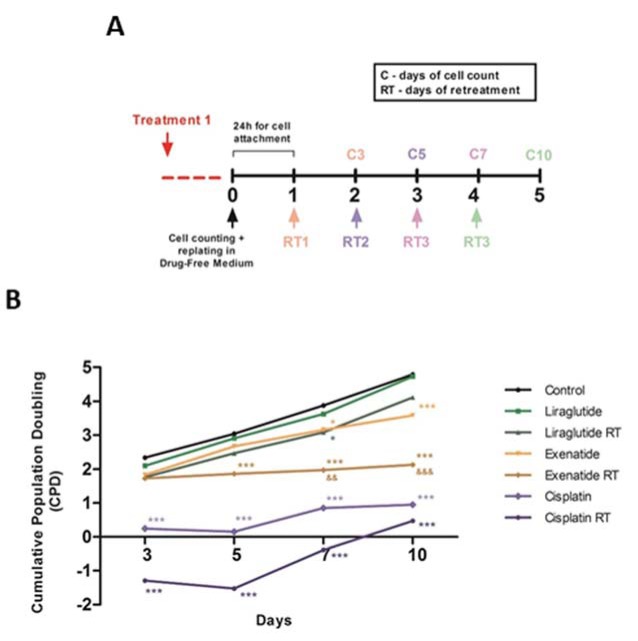
Exenatide reduces tumor cell regrowth. (A) Protocol of treatment. (B) Cells were exposed to liraglutide (15 μM), exenatide (15 μM) and cisplatin (20 μM positive control). Data represent the mean ± SD (*p < 0.05 vs control, ***p < 0.001 vs control) (^&&^ *p* < 0.01, ^&&&^ *p* < 0.001 vs liraglutide). RT represents retreatment.

Therefore, we decided to investigate the effects of long-term response of HepG2 cells after the treatment with exenatide and liraglutide, in single or multiple doses. The application of a single dose of exenatide did not suppress the regrowth of HepG2 cells, as well as both single and multiple doses of liraglutide treatment. However, multiple doses treatment with exenatide led to a stable arrest of the cell growth, indicating that exenatide may be a better long-term treatment for this tumor cell type (Figure 3A and 3B(Fig. 3)). 

## Discussion

GLP-1 exerts its role by binding to its specific receptor (GLP-1R) on human hepatocytes (Yoo et al., 2018[28]). Despite the controversy about the presence of these receptors in the liver, a recent study in human hepatoma cell lines revealed that exenatide has a dose-dependent effect in the increase of GLP-1R expression (Lee et al., 2012[16]). As an analog of GLP-1, which was first authorized to treat type 2 diabetes mellitus, exenatide can bind to the GLP-1R of pancreatic β-cells promoting the secretion of insulin. A clinical study with exenatide in type 2 diabetes showed a decreased hepatic fat accumulation, insulin resistance, and risk of cardiovascular diseases (Tushuizen et al., 2006[25]).

Several studies have shown that GLP-1 analogs may have anticancer potential. Chen et al. demonstrated an enhanced effect of chemotherapy in a mouse model of bile duct carcinoma treated with exendin-4 (Chen et al., 2013[4]). Moreover, experimental studies have shown that exendin-4 could inhibit and attenuates tumor growth (Honors and Kinzig, 2014[13]; Nomiyama et al., 2014[19]). Other studies revealed that GLP-1 analog liraglutide inhibits the growth of pancreatic cancer in animal models and hepatocellular carcinoma in HepG2 cell line (Zhao et al., 2014[29]; Krause et al., 2017[14]). These results corroborate our findings that exenatide decrease HepG2 cells viability after 48 h of treatment. In addition, the effect caused by exenatide demonstrates a greater potential for treatment than liraglutide, suggesting that exenatide may be considered as an important adjuvant in the treatment of HCC. Besides that, our results showed a low reduction of cell viability with a short treatment of 48 h.

Knowing that liraglutide is capable of inducing senescence in HepG2 cells (Krause et al., 2017[14]), we have tested this effect for exenatide. Senescence and apoptosis are two mechanisms that may be responsible for the proliferation decrease, as the first is characterized by an irreversible cell cycle arrest (Childs et al., 2014[5]) and the second by a programmed cell death with nuclear fragmentation (He et al., 2009[11]). While senescent cells adopt an expanded morphology, with flattened cytoplasm and increased granularity, the apoptotic cells suffer a high and regular condensation of the nucleus causing nuclear fragmentation (Saraste, 1999[22]). However, the treatment with exenatide did not show any morphological changes in the cellular nuclei, suggesting that there was no induction of senescence or apoptosis.

Another physiological cellular process involved in this mechanism is autophagy, which is one route of lysosome degradation that regulates cellular lipid metabolism and controls the innate immune activation, improving the state of insulin resistance and cancer (Dikic and Elazar, 2018[7]). Several works reported the beneficial effects of autophagy in carcinogenesis and studies of GLP-1 analogs have shown its ability to increase autophagy (He et al., 2016[12]; Li et al., 2017[17]). Here, we demonstrated, corroborating other studies, that exenatide is capable of inducing autophagy at a higher level than liraglutide, showing a superior potential and possibly justifying the decrease of cellular proliferation. To confirm the hypothesis that exenatide would be more efficient, we have also assessed how it would act on mTOR activation and inhibition. 

Autophagy is regulated by a complex signaling network and mTOR pathway is the main regulatory mechanism for its suppression (Paquette et al., 2018[20]). We observed that exenatide was able to inhibit insulin stimulatory effect in a more significant way than liraglutide, proving to have a greater effect. In addition, we demonstrated that liraglutide is able to inhibit mTOR protein expression in HepG2 cells, but exenatide causes a greater inhibition, justifying its greater induction of autophagy. 

Exenatide also showed antiproliferative effects in the treatment resistance analysis. The long-term evaluation and retreatment allowed the *in vitro* assay to be more comparable to clinical treatments, in which patients are submitted to many chemotherapeutic doses. This assay is clinically relevant, considering that chronic *in vitro* results are strongly associated with *in vivo* findings. (Augustine et al., 2009[2]). In our results, exenatide showed a greater potential to inhibit proliferation than liraglutide was able to maintain this effect over time.

We demonstrated for the first time that exenatide treatment does not allow the cells to grow again over time, one of the main problems experienced with the current chemotherapeutics, as shown for cisplatin. This resistance acquired by the cells makes the chemotherapy ineffective (Rebucci and Michiels, 2013[21]). Still, with exenatide, this resistance mechanism does not appear to occur, suggesting that this treatment may be an important finding in the search for new chemotherapeutics. 

Among the GLP-1 analogs tested, exenatide may be a better choice, as compared to liraglutide, for the HCC treatment. Furthermore, our results suggest that exenatide was able to attenuate the growth of HepG2 cells and the mTOR pathway may be the target of the underlying mechanism. However, further studies, both *in vivo* and clinical trials, should be employed in the search for the mechanisms in which exenatide is involved in the HCC, making this peptide a promising treatment for a disease of high incidence and mortality.

## Acknowledgement

G.C.K. was granted a fellowship from Coordenação de Aperfeiçoamento de Pessoal de Nível Superior (CAPES).

## Conflict of interest

The authors declare that they have no conflict of interest.
